# Miquelianin Inhibits Allergic Responses in Mice by Suppressing CD4^+^ T Cell Proliferation

**DOI:** 10.3390/antiox10071120

**Published:** 2021-07-13

**Authors:** Dae Woon Choi, Sun Young Jung, Gun-Dong Kim, So-Young Lee, Hee Soon Shin

**Affiliations:** 1 Food Biotechnology Program, Korea University of Science and Technology, Daejeon 34113, Korea; choidw19@gmail.com (D.W.C.); jsy5528@gmail.com (S.Y.J.); sylee09@kfri.re.kr (S.-Y.L.); 2 Division of Functional Food Research, Korea Food Research Institute, 245, Nongsaengmyeong-ro, Iseo-myeon 55365, Korea; kgd@kfri.re.kr

**Keywords:** atopic dermatitis, HO-1, miquelianin, trimellitic anhydride, Th2-related response

## Abstract

Allergic diseases, including atopic dermatitis (AD), induce type 2 helper T (Th2) cell-dominant immune responses. Miquelianin (quercetin 3-*O*-glucuronide, MQL) is an active compound in *Rosae multiflorae* fructus extract with anti-allergic properties. Here, we investigate the anti-allergic effects of MQL in an ovalbumin (OVA)-induced Th2-dominant mouse model and the associated mechanisms. Oral MQL suppressed cytokine and IL-2 production and proliferation of Th2 cells and upregulated heme oxygenase-1 (HO-1) in splenocytes. Ex vivo MQL suppressed Th1- and Th2-related immune responses by inhibiting CD4^+^ T cell proliferation, and upregulated HO-1 in CD4^+^ T cells by activating C-Raf–ERK1/2–Nrf2 pathway via induction of reactive oxygen species generation. In a trimellitic anhydride-induced AD-like mouse model, both topical and oral MQL ameliorated AD symptoms by suppressing Th2 immune responses. Our results suggest that MQL is a potential therapeutic agent for CD4^+^ T cell-mediated diseases, including allergic diseases.

## 1. Introduction

Allergic diseases, such as allergic rhinitis, asthma, food allergy, atopic dermatitis (AD), allergic contact dermatitis, and anaphylaxis, contribute to the rising cost of health care and result in a lower quality of life. Allergy is characterized by immune responses mediated by antigen-specific type 2 helper T (Th2) cells that are involved in the development of antigen-specific immunoglobulin E (IgE) responses produced following class switching of B cells [1]. T cell receptors (TCRs) in naïve CD4^+^ T cells recognize antigens upon co-stimulation by CD28 when contacting antigen-presenting cells (APCs). Antigen presentation to naïve CD4^+^ T cells induces their differentiation into effector and memory T cells and triggers clonal expansion. Activated T cells produce IL-2 that stimulates T cell proliferation via autocrine or paracrine signaling [2,3]. Naïve CD4^+^ T cells can differentiate into at least four subtypes, including Th1, Th2, Th17, and induced regulatory T (iTreg) cells [4]. Among these CD4^+^ T cells subsets, Th2 cells are characterized by their production of interleukin (IL)-4, IL-5, and IL-13 and stimulate B cell class switching to produce antigen-specific IgE [5].

Heme oxygenases (HOs) are essential enzymes that catabolize heme into iron (Fe), biliverdin, and carbon monoxide (CO). Three isoforms of HO exist: an inducible form (HO-1) and two constitutive forms (HO-2 and HO-3). The HO-1 isoform is an inducible enzyme expressed in mammalian tissues and plays a cytoprotective role [6,7,8]. Oxidative stress activates the nuclear factor erythroid 2-related factor 2 (NRF2) pathway resulting in HO-1 expression. In turn, HO-1 plays an essential role in maintaining intracellular homeostasis against excess reactive oxygen species (ROS) generated via redox signaling [9] and protects cells against oxidative stress and inflammation [10,11]. In addition, HO-1 can regulate cell proliferation by arresting the cell-cycle by generating CO [12,13]. Previous studies investigating the regulatory effects of HO-1 on T cells revealed that the CO generated by HO-1 also suppresses CD4^+^ T cell proliferation by inhibiting IL-2 production [14], and CO exposure suppresses proliferation and activation of T cells [15,16]. Phytochemicals obtained through fruit and vegetable consumption exert protective effects against diverse diseases by inducing HO-1 expression via activation of the NRF2 pathway [17]. Among these phytochemicals, quercetin, the aglycone of miquelianin (quercetin 3-*O*-glucuronide; MQL), has been reported to protect cells from oxidative stress and inflammation by generating HO-1 [18,19], and suppresses T cell proliferation via HO-1 generation [20].

We have previously shown that *Rosae multiflorae* fructus extract ameliorates OVA-induced allergic rhinitis symptoms in a mouse model and identified MQL as the active compound of *Rosae multiflorae* fructus [21]. MQL, a flavonoid, has been reported to have antioxidant [22], antidepressant [23], and antiproliferative properties [24]. However, its effects on allergic diseases and regulation of Th2 immune responses remains unknown. Thus, in this study, we examine the effect of MQL on a trimellitic anhydride (TMA)-induced AD-like mouse model and investigated the mechanisms by which MQL regulates Th2-related immune responses in vitro.

## 2. Materials and Methods

### 2.1. Materials

Imject^TM^ Alum Adjuvant was obtained from Pierce Biotechnology (Rockford, IL, USA). MitoSOX^TM^ Red was purchased from Thermo Fisher Scientific (Rockford, IL, USA). Mouse IL-2, IL-4, IL-5, IL-12, interferon (IFN)-γ, and IgE ELISA kits were purchased from BD Biosciences (San Diego, CA, USA). IL-13 ELISA kit was obtained from R&D Systems (Minneapolis, MN, USA). Specific primary antibodies for signal transducer and activator of transcription 6 (STAT6), p21, and β-actin were purchased from Santa Cruz Biotechnology (Santa Cruz, CA, USA). Primary antibody for phospho-STAT6 was obtained from Abcam (Cambridge, UK). Antibodies for phospho-STAT1, STAT1, phospho-STAT6, STAT6, phospho-ERK1/2, ERK1/2, phospho-p38, p38, phospho-JNK, JNK, NRF2, and HO-1 were purchased from Cell Signaling Technology (Danvers, MA, USA). Mouse anti-CD3 and anti-CD28 antibodies were purchased from Biolegend (San Diego, CA, USA). SP600125 was purchased from Calbiochem (San Diego, CA, USA). U0126 was obtained from Promega (Madison, WI, USA) and 2′,7′-Dichlorofluorescein-diacetate (DCFH-DA) was purchased from Invitrogen (Carlsbad, CA, USA). Propidium iodide (PI), SB203580, TMA, ovalbumin (OVA) (Grade VI), dexamethasone (Dex), concanavalin A (Con A), N-acetylcysteine (NAC), and MQL were purchased from Sigma-Aldrich (St. Louis, MO, USA).

### 2.2. Animals

Animals were maintained under specific pathogen-free conditions following established guidelines, and the experimental protocol was approved by the Animal Care and Use Committee of Korea Food Research Institute (KFRI-M-19003). Six-week-old female BALB/c mice were purchased from OrientBio Inc. (Gyeonggi-do, Korea) and housed in a temperature-controlled room (23 ± 2 °C) with a 12 h light/dark cycle and treated in accordance with the Korea Food Research Institute guidelines for animal care and use.

### 2.3. Induction of AD-Like Symptoms by TMA

We generated a TMA-induced AD-like mouse model by modifying previously reported methods [25,26]. To evaluate the anti-AD effects of topical MQL treatment, the BALB/c mice were divided into five groups, namely naïve (TMA-non-exposed, *n* = 6), sham (untreated, *n* = 6), MQL4 (4 mg/mL MQL-treated, *n* = 6), MQL10 (10 mg/mL MQL-treated, *n* = 6), and Dex (1 mg/mL Dex-treated, *n* = 6). Mice were sensitized on the shaved right flank with 50 μL of 5% TMA in a mixed solvent (acetone/isopropyl myristate, 4:1 *v/v*) on day 0, and then 10 μL of 2% TMA was applied to the surface of both ears daily from day 5 to 14. MQL or Dex (10 μL) was administered topically daily from day 5 to 14, then mice were sacrificed on day 15.

To evaluate the anti-AD effects of oral MQL treatment, the BALB/c mice were divided into five groups: naïve (TMA-non-exposed, *n* = 6), sham (untreated, *n* = 8), MQL4 (4 mg/mL MQL-treated, *n* = 8), MQL10 (10 mg/mL MQL-treated, *n* = 8), and Dex (1 mg/mL Dex-treated, *n* = 6). Mice were sensitized on the shaved right flank with 50 μL of 5% TMA in a mixed solvent (acetone/isopropyl myristate, 4:1 *v/v*) on day 0, and then 20 μL of 2% TMA was applied to the surface of both ears on days 5, 8, 11, 14, 17, 20, 23, and 26. MQL or Dex was administered orally daily from day 5 to 26. Ear thickness was measured at 24 h after TMA treatment using a custom-built micrometer (Schering AG, Berlin, Germany). Mice were sacrificed on day 27.

### 2.4. OVA-Induced Allergic Mouse Model

To evaluate the effects of MQL on allergic immune responses in vivo, we used a mouse model of allergy induced by i.p. injection of OVA and alum. The BALB/c mice were divided into five groups: naive (OVA non-injected, *n* = 6), sham (untreated, *n* = 8), MQL2 (2 mg/kg MQL-treated, *n* = 8), MQL4 (4 mg/kg MQL-treated, *n* = 8), and Dex (1 mg/kg Dex-treated, *n* = 6) groups, and sensitized by i.p. injection with 10 μg OVA adsorbed to 1 mg of alum on days 0 and 14. MQL was orally administered daily from day 7 to 21, and the mice in naive and sham groups were administered with saline instead of MQL. Mice were sacrificed on day 22.

### 2.5. Culture of Draining Lymph Node (DLN) Cells and Splenocytes

DLNs isolated from the TMA-induced AD mice were prepared by aseptically removing and then homogenizing the tissues from each mouse to single cells. DLN cells were cultured to 1 × 10^6^ cells/mL in RPMI 1640 medium (containing 10% fetal bovine serum, 100 U/mL penicillin, and 100 mg/mL streptomycin) with Con A (2 μg/mL) for 48 h. Splenocytes isolated from OVA-induced AD mice and allergic mouse models were cultured to 5 × 10^6^ cells/mL in RPMI 1640 medium (containing 10% fetal bovine serum, 100 U/mL penicillin, and 100 mg/mL streptomycin) with OVA (100 μg/mL) for 72 h. Cells were incubated at 37 °C in a humidified incubator with 5% (*v/v*) CO_2_ and 95% (*v/v*) air.

### 2.6. In Vitro Assay Using Mouse Splenocytes Immunized by OVA

BALB/c mice were sensitized by i.p. injection with 10 μg OVA adsorbed in 1 mg of alum on days 0 and 14, then mice were sacrificed on day 21. Splenocytes isolated from mice immunized by OVA were cultured to 5 × 10^6^ cells/mL in RPMI 1640 medium (containing 10% fetal bovine serum, 100 U/mL penicillin, and 100 mg/mL streptomycin) with OVA (100 μg/mL) and MQL for 72 h. Cells were incubated at 37 °C in a humidified incubator with 5% (*v/v*) CO_2_ and 95% (*v/v*) air.

### 2.7. CD4^+^ T Cell Isolation and Proliferation Assay

CD4^+^ T cells were isolated from BALB/c mouse splenocytes using the Magnisort^TM^ mouse enrichment kit (Invitrogen). Purified CD4^+^ T cells (1 × 10^6^ cells/mL) were activated after seeding them in 96-well plates pre-coated with anti-CD3 (1 μg/mL) and anti-CD28 antibodies (1 μg/mL) in the presence or absence of MQL. After 2 days, CD4^+^ T cell proliferation was assessed by MTT assay.

### 2.8. HO-1 and NRF2 Detection in CD4^+^ T Cells

CD4^+^ T cells were activated after seeding them in 96-well plates pre-coated with anti-CD3 (1 μg/mL) and anti-CD28 antibodies (1 μg/mL) in the presence or absence of the sample. To analyze the mitogen-activated protein kinases (MAPKs) involved in MQL-induced HO-1 expression, cells were pre-treated with U0126 (ERK inhibitor, 5 μM), SB203580 (p38 inhibitor, 5 μM), or SP600125 (JNK inhibitor, 5 μM) for 1 h before sample treatment.

To detect Nrf2, the cytoplasm and nuclei of cultured CD4^+^ T cells were separated using the NE-PER Nuclear and Cytoplasmic Extraction Reagent kit (Cell Signaling Technology, Danvers, MA, USA). HO-1 and Nrf2 levels were detected by Western blot.

### 2.9. Cell Cycle Analysis

To confirm the effects of MQL on the cell cycle in CD4^+^ T cells, we conducted PI fluorescence assays. Briefly, harvested CD4^+^ T cells in the presence or absence of MQL were fixed in cold 70% ethanol at 4 °C. After 30 min, cells were centrifuged at 85× *g* and washed in PBS. Next, cells were treated with ribonuclease (100 μg/mL, 50 μL) and PI solution (50 μg/mL, 200 μg/mL). The presence of single cells was confirmed by flow cytometry, using a CytoFLEX analyzer (Beckman Coulter, Pasadena, CA, USA).

### 2.10. ROS Detection

DCFH-DA was used to detect ROS and MitoSOX^TM^ Red was used to detect mitochondrial superoxide. CD4^+^ T cells (1 × 10^6^ cells/mL) were treated with MQL (50 μg/mL) for 1 or 2 h and washed with HBSS/Ca^2+^/Mg^2+^. CD4^+^ T cells were then incubated for 10 min in HBSS/Ca^2+^/Mg^2+^ buffer containing DCFH-DA or MitoSOX^TM^ Red (5 μM). After staining, cells were washed and ROS levels were detected using a fluorescence plate reader (SpectraMax^®^ i3, Molecular Devices, San Jose, CA, USA; DCFH-DA-Ex/Em: 490/535 nm, MitoSOX^TM^ Red-Ex/Em: 510/580 nm) and flow cytometry.

### 2.11. Measurement of Cytokines and Serum IgE Using ELISA

The levels of cytokines (IL-2, IL-4, IL-5, IL-12, IL-13, and IFN-γ) and IgE were quantified using ELISA kits according to the manufacturer’s protocols. Briefly, sample and standard solutions were transferred to 96-well plates pre-coated with the appropriate monoclonal antibodies, and then incubated at room temperature for 2 h. After thorough washing, horseradish peroxidase-conjugated secondary antibodies were added to each well, then incubated at room temperature for 1 h. After removal of the secondary antibodies, the substrate solution was added, and samples were incubated for another 30 min in the dark. The reaction was terminated by addition of stop solution, and absorbance was measured at 450 nm using a microplate reader (BioTek, Winooski, VT, USA).

### 2.12. Western Blotting

To investigate the effects of MQL on the phosphorylation of STAT1, STAT6, and HO-1, splenocytes (5 × 10^6^ cells/mL) isolated from mice immunized by OVA were cultured with OVA (100 μg/mL) and MQL for 72 h. The phosphorylation of ERK1/2, p38, JNK, and C-Raf were detected in CD4^+^ T cells cultured for 2 h with MQL. Protein levels were measured using an automated capillary-based size sorting system (WES; ProteinSimple, Santa Clara, CA, USA). All procedures were performed according to the manufacturer’s instructions and the data were analyzed with Compass software (ProteinSimple).

### 2.13. Statistical Analysis

Data are expressed as the mean ± standard deviation (SD), and significant differences were determined using one-way ANOVA (analysis of variance). A *p*-value < 0.05 was used as the minimum threshold for determining statistical significance.

## 3. Results

### 3.1. Effect of Oral MQL Administration on OVA-Induced Allergy

**Figure 1:**
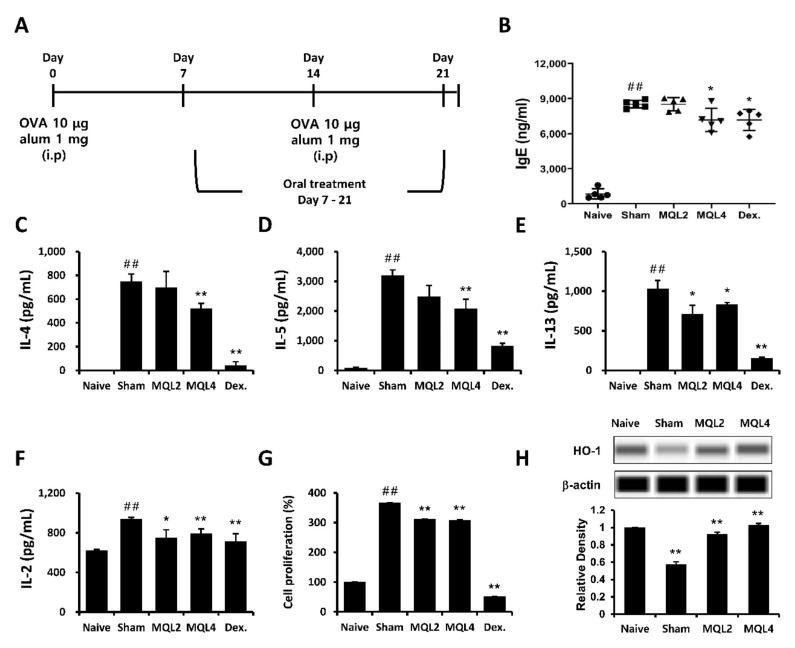
Effects of MQL on the OVA-induced allergic mouse model. (**A**) Experimental schedule of the OVA-induced allergic mouse model. Allergic immune responses in BALB/c mice were induced by i.p. injection of OVA (10 μg) with alum (1 mg) on days 7 and 14. MQL was orally administered daily from days 7 to 21. (**B**) The level of serum IgE was analyzed using ELISA. (**C**–**F**) Splenocytes were seeded to 5 × 10^6^ cells/mL and cultured in the presence of OVA (100 μg/mL) for 72 h. (**C**) IL-4, (**D**) IL-5, (**E**) IL-13, and (**F**) IL-2 cytokines in culture supernatant of splenocytes were measured using ELISA. (**G**) Cell proliferation was measured using MTT assay, and (**H**) HO-1 expression was detected in splenocytes of each group using Western blot. Results are shown as mean ± SD. ## *p* < 0.01, between the naïve and sham groups, * *p* < 0.05, ** *p* < 0.01, between the MQL-treated and sham groups.

In this study, we examined whether oral administration of MQL affects Th2-related allergic immune responses using an OVA-induced mouse model of allergy. The mice were sensitized and immunized by OVA and orally administered MQL for 2 weeks (Figure 1A). We investigated typical allergic responses such as production of IgE and Th2-mediated cytokines including IL-4, IL-5, and IL-13. We found that levels of serum IgE and splenic Th2 cytokines were significantly suppressed by oral administration of MQL (Figure 1B–E).

In our previous study, we showed that *Rosae multiflorae* fructus extract containing MQL suppressed Th2-related immune responses by inhibiting the proliferation of immune cells via up-regulation of HO-1 expression. Similarly, it has been reported that increased HO-1 expression in CD4^+^ T cells suppressed proliferation by inhibiting IL-2 production [7]. Based on these studies, we measured IL-2 production and proliferation of splenocytes and confirmed that these were reduced in MQL-administered groups compared to the sham group (Figure 1F,G). Furthermore, we found that splenocytes in MQL-administered groups had increased HO-1 expression compared with those in the sham group (Figure 1H).

### 3.2. Immunomodulatory Effects of MQL on Splenocytes from BALB/c Mice Immunized by OVA

Splenocytes from BALB/c mice immunized twice with OVA and alum were isolated. Using this ex vivo system, we investigated whether MQL treatment regulates allergic immune responses by suppressing cell proliferation via increased HO-1 expression.

**Figure 2:**
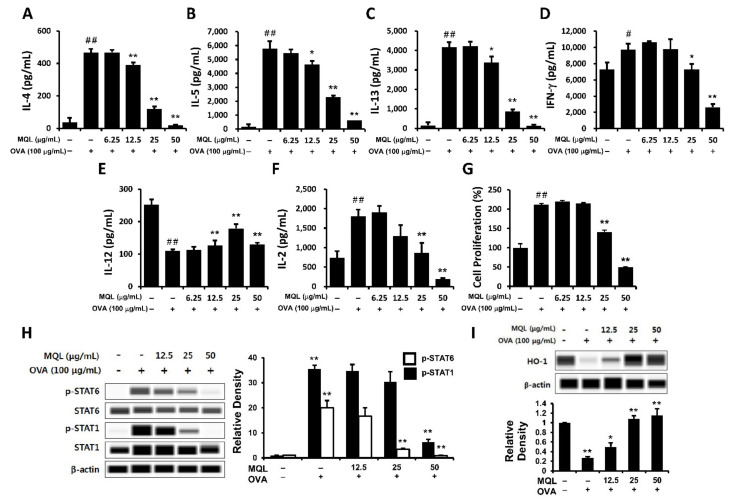
Immunomodulatory effects of MQL on splenocytes of BALB/c mice immunized by OVA. Allergic immune responses in BALB/c mice were induced by i.p. injection of OVA (10 μg) with alum (1 mg) on days 7 and 14. Splenocytes were seeded to 5 × 10^6^ cells/mL and cultured in the presence or absence of OVA (100 μg/mL) and MQL for 72 h. (**A**) IL-4, (**B**) IL-5, (**C**) IL-13, (**D**) IFN-γ, (**E**) IL-12, and (**F**) IL-2 cytokines in culture supernatant of splenocytes were measured using ELISA. (**G**) Cell proliferation was measured using MTT assay. (**H**) STAT1 and STAT6 phosphorylation and (**I**) HO-1 expression were detected using Western blot. Results are shown as mean ± SD. # *p* < 0.05, ## *p* < 0.01, between the non-treated and OVA-treated groups, * *p* < 0.05, ** *p* < 0.01, between the MQL-treated and OVA-treated groups.

OVA-induced Th1- (IFN-γ) and Th2-related cytokines (IL-4, IL-5, and IL-13) were significantly suppressed by MQL treatment (Figure 2A–D). Next, to investigate whether the immunomodulatory effects of MQL were a consequence of decreased cell proliferation, we measured proliferation and IL-2 production. Notably, MQL treatment suppressed IL-2 production as well as splenocyte proliferation (Figure 2F,G). To further characterize these T cell subsets, we analyzed Th1- (STAT1) and Th2-associated transcriptional factors (STAT6) as well as HO-1. Figure 2H,I show that STAT6 and STAT1 phosphorylation were reduced by MQL treatment, whereas HO-1 expression was increased by MQL. Interestingly, the Th1-related cytokine IL-12 was increased by MQL treatment (Figure 2E). These results indicate that MQL treatment largely inhibits Th1- and Th2-related immune responses via reduction of cell proliferation induced by HO-1 up-regulation.

### 3.3. Effects of MQL on CD4^+^ T Cell Proliferation and HO-1 Expression

**Figure 3:**
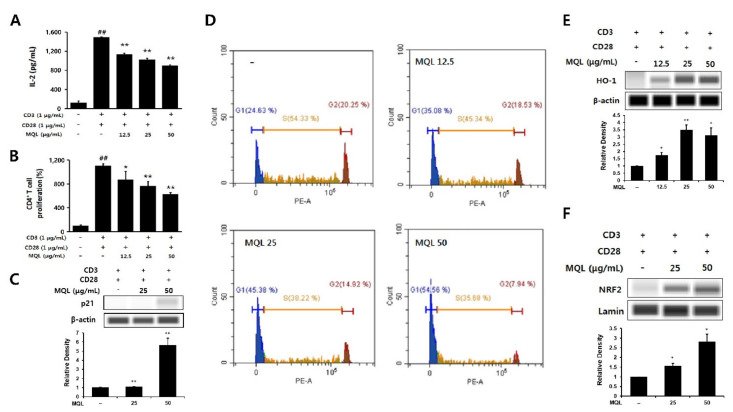
Effects of MQL on CD4^+^ T cell proliferation and HO-1 expression. CD4^+^ T cells (1 × 10^6^ cells/mL) isolated from splenocytes were seeded and cultured with anti-CD3 (1 μg/mL) and anti-CD28 antibodies (1 μg/mL). CD4^+^ T cells were cultured in the presence or absence of MQL for 48 h. (**A**) IL-2 in culture supernatant of CD4^+^ T cells was measured using ELISA. (**B**) CD4^+^ T cell proliferation was measured using MTT assay. (**C**) Expression of p21 was detected using Western blot, and (**D**) cell cycle of CD4^+^ T cells was measured using flow cytometry. (**E**) HO-1 expression and (**F**) NRF2 nuclear translocation were detected in CD4^+^ T cell cultured in the presence or absence of MQL using Western blot. Results are shown as mean ± SD. ## *p* < 0.01, between the non-stimulated and CD3/28-stimulated groups, * *p* < 0.05, ** *p* < 0.01, between the MQL-treated and CD3/28-stimulated groups.

IL-2 is an essential factor for CD4^+^ T cell proliferation and is produced following antigen recognition by TCR with co-stimulation by CD28. IL-2 then binds its receptor via paracrine and autocrine mechanisms and causes clonal expansion of CD4^+^ T cells [3]. In this experiment, naïve CD4^+^ T cells were isolated and stimulated with anti-CD3 (TCR stimulation) and anti-CD28 antibodies (co-stimulation). We then investigated the effects of MQL on CD4^+^ T cell proliferation and IL-2 production and found that MQL treatment suppressed both proliferation and IL-2 production in a dose-dependent manner (Figure 3A,B).

p21 (known as p21^WAF1/Cip1^) is a cyclin-dependent kinase (CDK) inhibitor and arrests cell cycle progression in the G1 phase [27]. Up-regulation of HO-1 was reported to increase p21 levels, thus reducing cell proliferation. Therefore, we investigated whether MQL affects the cell cycle in CD4^+^ T cells and observed that MQL treatment increased p21 levels and arrested the cell cycle at the G1 stage compared with untreated CD4^+^ T cells (Figure 3C,D).

Nrf2 is a transcription factor of HO-1 that is sequestered in the cytoplasm by binding with Kelch-like ECH-associated protein 1 (Keap1). When Nrf2 is released from Keap1, it translocates into the nucleus and increases transcription of the HO-1 gene by binding to its antioxidant response element (ARE) [28,29]. We therefore investigated Nrf2 translocalization following MQL treatment and found that NRF2 nuclear translocation and consequent HO-1 expression were increased by MQL in CD4^+^ T cells (Figure 3E,F). Collectively, these results demonstrate that MQL upregulates p21, arrests the cell cycle, and activates the NRF2–HO-1 pathway, resulting in reduced CD4^+^ T cell proliferation.

### 3.4. Effects of MQL on MAPK Signaling Pathways

**Figure 4:**
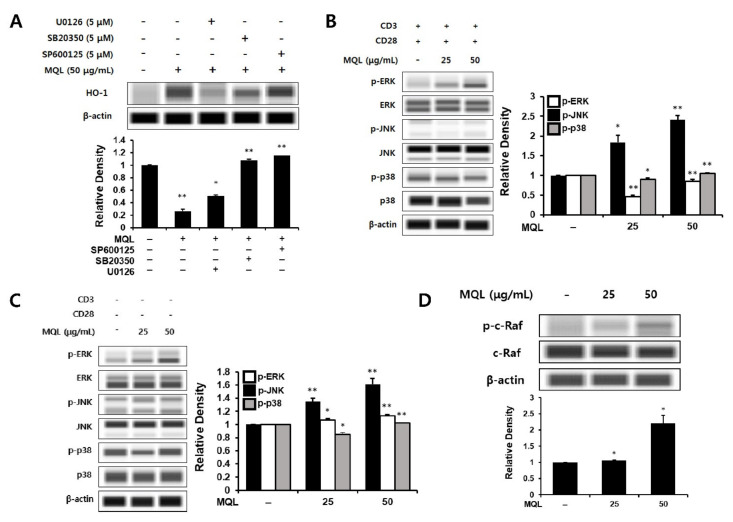
Effects of MQL on MAPK signaling pathways. (**A**) CD4^+^ T cells were isolated from splenocytes and pre-treated with U0126 (5 μM), SB20350 (5 μM), or SP600125 (5 μM) for 1 h and then treated with MQL for 24 h to detect HO-1 expression in CD4^+^ T cells. (**B**) To analyze MAPK phosphorylation induced by MQL, CD4^+^ T cells were pre-treated with MQL for 2 h before stimulation with anti-CD3 (1 μg/mL) and anti-CD28 antibodies (1 μg/mL). CD4^+^ T cells were treated with MQL for 2 h without stimulation, and (**C**) MAPK phosphorylation and (**D**) C-Raf phosphorylation were detected using Western blot. Data are representative of three independent experiments. Results are shown as mean ± SD. * *p* < 0.05, ** *p* < 0.01, between the MQL-treated and non-treated groups.

Next, to investigate MAPKs upstream of MQL-induced HO-1 expression, we cultured CD4^+^ T cells with U0126 (ERK inhibitor), SB20350 (p38 inhibitor), or SP600125 (JNK inhibitor) in the presence of MQL. We found that MQL-induced HO-1 expression was most significantly reduced by U0126 treatment compared with the other inhibitors (Figure 4A). To explore this further, we measured ERK1/2, p38, and JNK activation in the presence or absence of T cell stimulation (anti-CD3/CD28 antibodies). Consistent with the previous finding, MQL dose-dependently increased the phosphorylation of ERK, but not p38 or JNK (Figure 4B,C).

The Ras-Raf-MEK-ERK signaling pathway plays a crucial role in gene expression related to cell growth, proliferation, differentiation, and survival [30,31]. Ras proteins are small GTPases that can activate Raf-MEK-ERK signaling [32]. GTP-bound Ras interacts with the Raf family (A-Raf, B-Raf, and C-Raf), inducing Raf dimerization and phosphorylation of C-Raf (also called Raf-1) [33,34]. It has also been reported that phosphorylation of C-Raf (Ser338) can induce ERK activation in lymphocytes such as T cells and B cells [35]. Therefore, we hypothesized that C-Raf could be the signal upstream of ERK, and indeed found that MQL treatment induced phosphorylation of C-Raf (Ser338) (Figure 4D).

### 3.5. Effects of MQL on ROS Generation in CD4^+^ T Cells

**Figure 5:**
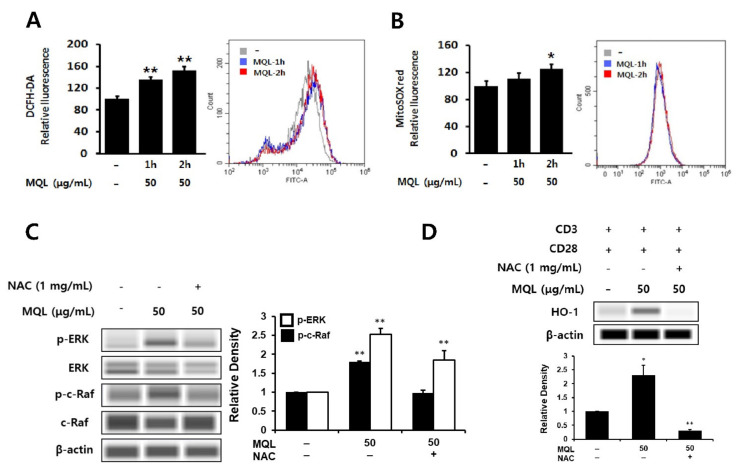
Effects of MQL on ROS generation in CD4^+^ T cells. CD4^+^ T cells isolated from splenocytes were cultured in the presence or absence of MQL for 2 h. Production of reactive oxygen species in CD4^+^ T cells was detected using (**A**) DCFH-DA and (**B**) MitoSox^TM^ Red and measured using a fluorescence plate reader or flow cytometry. (**C**) CD4^+^ T cells isolated from splenocytes were pre-treated with NAC (1 mg/mL) for 1 h, then cultured for 2 h in the presence of MQL. Phosphorylation of ERK and C-Raf were detected using Western blot. (**D**) CD4^+^ T cells were cultured with anti-CD3 (1 μg/mL) and anti-CD28 antibodies (1 μg/mL) for 24 h in the presence of NAC (1 mg/mL) and MQL. HO-1 expression in CD4^+^ T cells was detected using Western blot. Results are shown as mean ± SD. * *p* < 0.05, ** *p* < 0.01, between the MQL-treated and non-treated groups.

Phytochemicals have been well-known as antioxidants that can directly scavenge ROS and are used to protect against inflammation [36,37]. However, phytochemicals can beneficially induce the generation of intracellular ROS, resulting in up-regulation of HO-1 expression [38]. Furthermore, intracellular ROS have been linked to ERK1/2 as cAMT-mediated ROS production can induce ERK1/2 phosphorylation via Ras activation [39]. Hence, we investigated intracellular ROS generated by MQL in CD4^+^ T cells using DCFH-DA or MitoSOX^TM^ Red and found that MQL increased intracellular and mitochondrial ROS levels (Figure 5A,B). Next, to investigate the effects of intracellular ROS generated by MQL on the C-Raf-ERK1/2 signaling pathway, CD4^+^ T cells were cultured with MQL in the presence and absence of the antioxidant NAC. We found that MQL-induced C-Raf and ERK1/2 phosphorylation as well as HO-1 expression were suppressed by NAC treatment (Figure 5C,D). Taken together, these results suggest that MQL-induced ROS mediate Ras-Raf-ERK activation and HO-1 expression in CD4^+^ T cells.

### 3.6. Effects of Topical and Oral MQL Administration on TMA-Induced AD Symptoms

**Figure 6:**
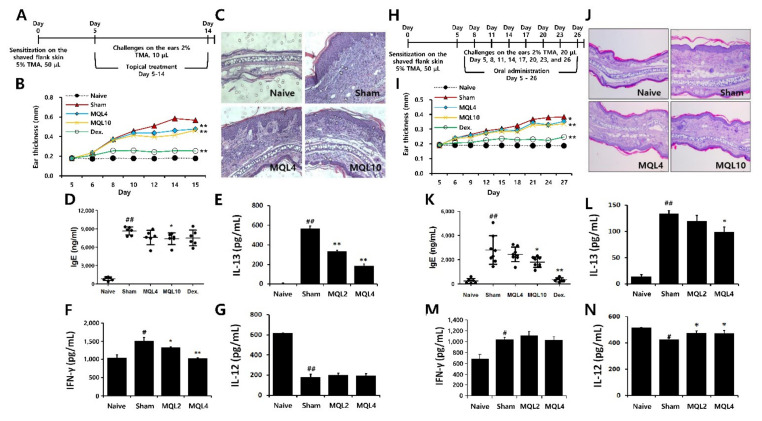
Effects of topical and oral administration on TMA-induced AD symptoms. (**A**) Experimental schedule of the TMA-induced AD-like mouse model with topical treatment of MQL. Cells from DLNs (1 × 10^6^ cells/mL) were seeded and cultured in the presence of Con A (2 μg/mL) for 48 h. An experimental AD-like lesion was induced on the shaved flank skin of BALB/c mice by sensitization with 5% TMA (50 μL) on day 0, followed by treatment with 2% TMA (10 μL) on days 5 to 14. MQL (4 and 10 mg/mL, 10 μL) and Dex (1 mg/mL, 10 μL) were administered topically. (**B**) Ear swelling was measured 24 h after TMA treatment. (**C**) Infiltrating inflammatory cells in the ear tissues were stained using hematoxylin and eosin. The levels of (**D**) IgE in serum and (**E**) IL-13, (**F**) IFN-γ, and (**G**) IL-12 cytokines in culture supernatant of DLNs were measured using ELISA. (**H**) Experimental schedule of the TMA-induced AD-like mouse model with oral administration of MQL. An experimental AD-like lesion was induced on the shaved flank skin of BALB/c mice by sensitization with 5% TMA (50 μL) on day 0 then treated with 2% TMA (20 μL) on days 5, 8, 11, 14, 17, 20, 23, and 26. MQL (4 and 10 mg/kg) and Dex (1 mg/kg) were administrated by orally from day 5 to day 26. (**I**) Ear swelling was measured 24 h after TMA treatment. (**J**) Infiltrating inflammatory cells in the ear tissues were stained using hematoxylin and eosin. The levels of (**K**) IgE in serum and (**L**) IL-13, (**M**) IFN-γ, and (**N**) IL-12 cytokines in culture supernatant of DLNs were measured using ELISA. Results are shown as mean ± SD. # *p* < 0.05, ## *p* < 0.01, between the naïve and sham groups, * *p* < 0.05, ** *p* < 0.01, between the MQL-treated and sham groups.

To evaluate the effects of MQL on Th2-mediated allergic diseases, we used a TMA-induced AD-like mouse model. Since AD symptoms have been known to occur through both external and internal factors, we administered MQL treatment either topically or orally. After topical treatment of MQL, ear thickness and infiltration of inflammatory cells were reduced compared to the sham group (Figure 6A–C). Furthermore, serum IgE levels were reduced in MQL10 group (Figure 6D). Next, to investigate the effects of MQL on T cell-mediated immune responses, we measured Th1 (IL-12 and IFN-γ) and Th2 (IL-13) cytokine production in the culture supernatant of DLNs and found that IL-13 and IFN-γ production were significantly reduced by MQL treatment, but that of IL-12 was not changed (Figure 6E–G).

Next, we investigated effect of oral MQL administration on TMA-induced AD. Similar to topical administration, oral MQL administration ameliorated AD symptoms such as ear swelling and infiltration of inflammatory cells in ear (Figure 6H–J), and TMA-induced serum IgE production was decreased in the MQL-administered group compared with that in the sham group (Figure 6K). In DLNs, oral administration of MQL reduced IL-13 production and recovered the diminished IL-12 levels compared to the sham group (Figure 6L,N). These results suggest that either topical or oral administration of MQL is a possible treatment to attenuate the symptoms of AD.

## 4. Discussion

To investigate the effects of MQL on allergic immune responses, we used an OVA-induced allergic mouse model, and showed that MQL suppresses Th2-related immune responses such as serum IgE production and Th2 cytokine production by splenocytes. We have previously shown that MQL is one of the active compounds in *Rosae multiflorae* fructus extract that can ameliorate asthma, rhinitis, and food allergies [21,40]. Furthermore, Nguyen et al. reported that *Rosae multiflorae* fructus exerts an anti-allergic effect by suppressing antigen-specific T cell activation and proliferation in vivo [41]. Our in vivo study results also suggest that MQL suppresses splenocyte proliferation and IL-2 production, thereby inhibiting Th2-related immune responses. Therefore, we focused on CD4^+^ T cells and investigated the anti-allergic mechanisms of MQL.

Th2-dominant responses, with increased Th2 (IL-4, IL-5, and IL-13) cytokines and IgE production, are characteristic immune responses in patients with allergic diseases such as food allergy, asthma, and AD [42,43,44]. We previously reported a few strategies to treat allergic diseases [45,46,47]. The first involves regulating the Th1/Th2 immune balance by suppressing excessively induced Th2-mediated immune responses or increasing Th1-related immune responses [48,49,50]. However, we confirmed that MQL treatment does not increase the Th1-associated cytokine IFN-γ. Thus, we focused on T cell proliferation as reducing T cell clonal expansion and differentiation represents an alternative strategy to manage allergic diseases [2]. Mechanisms for suppressing T cell proliferation typically include inducing regulatory T cells (Treg) or up-regulating HO-1 expression. In our investigation of the effects of MQL on Treg induction, MQL treatment did not increase CD4^+^Foxp3^+^ T cell populations (data not shown). Therefore, in the present study, we investigated the effects of MQL on Th2-dominant allergic immune responses and mechanisms involved in HO-1 expression.

HO-1 has various physiological functions including a protective effect against oxidative stress [12,13]. In particular, it was reported that up-regulation of HO-1 in CD4^+^ T cells suppresses their proliferation by inhibiting IL-2 production [7]. Similarly, our results showed that MQL suppresses CD4^+^ T cell proliferation and IL-2 production induced by anti-CD3/CD28 antibody stimulation, while HO-1 expression was increased. Based on these results, we hypothesize that MQL may be able to suppress the CD4^+^ T cell-mediated immune responses by inhibiting proliferation of CD4^+^ T cells via up-regulation of HO-1. Furthermore, we speculate that HO-1 is a key regulator of CD4^+^ T cell proliferation.

Upstream signals of HO-1 have been previously reported. Notably, Nrf2, which is normally repressed by Keap1, translocates to the nucleus upon activation and binds to ARE sequences of the HO-1 gene. In particular, activation of MAPKs (ERK, JNK, and p38) as up-stream signals of Nfr2 plays a role in the regulatory effect of HO-1 on cell proliferation. However, the specific MAPKs involved depend on the administered compounds. For example, quercetin enhances Nrf2/HO-1 activity via both ERK and p38 signaling pathways [51,52] and suppresses T cell proliferation by enhancing HO-1 expression [20]. Similarly, quercetin-3-*O*-*β*-D-glucuronopyranoside, also known as isoquercitrin, can up-regulate HO-1 expression via ERK pathways [53]. In a recent study, Lee et al. revealed that MQL up-regulates HO-1 expression via the Nrf2 pathway in human hepatoma cells (HepG2), but does not activate nuclear factor-kappa B (NF-κB) [54]. In this study, we found that MQL activated Nrf2-HO-1 signals through phosphorylation of ERK in CD4^+^ T cells. In experiments using MAPK inhibitors, we confirmed that U0126 (ERK inhibitor) most significantly suppressed HO-1 expression, and MQL strongly increased ERK phosphorylation in CD4^+^ T cells. Although treatment with SB20350 (p38 inhibitor) suppressed HO-1 expression, it was relatively weak compared with the effect of U0126. Moreover, p38 phosphorylation was not detected following MQL treatment. Therefore, our results demonstrate that ERK activation is upstream of MQL-induced HO-1 expression in CD4^+^ T cells.

Ras is a small GTP-binding protein with three isoforms (H-Ras, K-Ras, and N-Ras) and controls growth via ERK activation and other intracellular signaling pathways [55]. Membrane-bound Ras (GTP-bound active form) can activate Raf by recruiting Raf and promoting the formation of B-Raf/C-Raf complexes or homodimers. Activated Raf protein can then induce phosphorylation of ERK1/2 [34,56]. Previous studies have reported that HO-1 expression can be up-regulated by activating the Ras-Raf-ERK signaling pathway [57,58]. Based on these findings, we investigated whether MQL-induced HO-1 up-regulation was associated with C-Raf phosphorylation and found that MQL could induce phosphorylation of C-Raf in CD4^+^ T cells, indicating that C-Raf could be a potential up-stream signal of the ERK-Nrf2 signaling pathway.

ROS such as superoxide anion radicals (O_2_^•−^), hydroxyl radicals (•OH), and hydrogen peroxide (H_2_O_2_) are constantly produced as byproducts of mitochondrial oxidative metabolism. Excessively generated ROS, however, can induce oxidative stress, which underlies various diseases, while normal ROS levels can regulate cell growth by modulating proliferation and differentiation. Specifically, ROS activate cellular pathways such as MAPK, JAK/STAT, Nrf2, and NF-κB signals [59,60,61]. Furthermore, intracellular ROS are known to induce HO-1 expression [62,63]. Consistent with our results, intracellular ROS has been reported to activate the Raf-MEK-ERK signaling pathways [64] and induce ERK1/2 phosphorylation by activating Ras [39,65]. Importantly, flavonoids are antioxidant secondary phenolic metabolites naturally produced in fruits and vegetables and are able to offer protection against oxidative stress [66]. However, flavonoids can also increase intracellular ROS levels as prooxidants. For example, McNally et al. revealed that curcumin increases HO-1 expression via ROS generation [67]. In addition, it has been reported that quercetin can also act as a prooxidant capable of producing intracellular ROS [37,68]. In this study, we found that MQL induces ROS production in CD4^+^ T cells, which enhances HO-1 expression via Raf-ERK activation. Consequently, this leads to suppression of IL-2 production and CD4^+^ T cell proliferation.

TMA, as a hapten, can induce allergic AD by evoking Th2-dominant immune responses in mice [29,69,70,71]. Haptens act as antigens by generating hapten–protein complexes through conjugation with self-proteins and then activate adaptive immune responses when recognized by APCs such as dendritic cells (DCs) [71]. Langerhans cells, which are DCs that reside in the epidermis, induce naïve CD4^+^ T cell differentiation by presenting antigens after migrating into T cell-rich areas, such as the paracortex of the DLNs [72]. To characterize the effects of MQL on a TMA-induced AD-like mouse model, we administered MQL either orally or topically, since both oral and topical therapies are effective for treating AD. Considering that this was a disease model, we used a higher dosage of MQL (4 and 10 mg/kg) than that used in the OVA-induced Th2-dominant mouse model (2 and 4 mg/kg). Our results revealed that AD symptoms, such as ear swelling, tissue infiltration of inflammatory cells, and IgE production, were ameliorated by MQL in both topical and oral treatments. Interestingly, serum IgE levels were strongly reduced by oral administration of MQL compared with topical administration, possibly because IgE production involves a systemic immune response. However, in DLNs, which represent peripheral immune responses, IL-13 levels induced by TMA were dramatically reduced by topical treatment of MQL compared with oral administration. These results indicate that MQL may be effective as an oral or topical drug depending on the internal or external causes of atopic dermatitis. Further anti-proliferative effects of MQL were seen in allergic mouse models as Th2 (IL-13) and Th1 (IFN-γ) cytokines decreased, but not IL-12 (cytokine derived from APC). Furthermore, IFN-γ was suppressed by MQL topical treatment, but not IL-12, while oral administration of MQL did not affect IFN-γ production but increased IL-12 production. More evidently, IFN-γ/IL-12 ratios showed that MQL treatment decreased IFN-γ production compared with the TMA alone group as follows: Ratio of IFN-γ/IL-12 in the naïve group (oral, 1.3; topical, 1.7), TMA-induced AD group (oral, 2.4; topical, 8.9), MQL 4 group (oral, 2.3; topical, 6.5), and MQL 10 group (oral, 2.2; topical, 5.3). Therefore, we believe that MQL has the potential to be developed as a therapeutic agent for AD.

## 5. Conclusions

In this study, we demonstrated that MQL suppressed Th2-related immune responses. MQL suppresses OVA and TMA-induced inflammatory gene expression including IL-2, IL-4, IL-5, IL-12, IL-13, and IFN-γ by reducing CD4^+^ T cell proliferation via up-regulation of HO-1 expression. Mechanistically, MQL increased HO-1 expression via activation of the Raf-ERK-Nrf2 pathway and by generating ROS in CD4^+^ T cells. Furthermore, we verified that these effects of MQL on CD4^+^ T cells lead to alleviated allergic diseases such as an atopic dermatitis in vivo. MQL attenuates AD-like clinical symptoms including IgE hyper production, dermal thickening, swelling, and recruitments of inflammatory cells in mice. It is possible that MQL may provide clinical benefits in other allergic and CD4^+^ T cell-mediated diseases. The application of MQL on various diseases remains the focus of further studies.

## Data Availability

All the data analyzed for this manuscript are included. The analyzed raw data are available upon reasonable request to the corresponding author.
